# Fibroblast growth factor 23 and parathyroid hormone after treatment with active vitamin D and sevelamer carbonate in patients with chronic kidney disease stage 3b, a randomized crossover trial

**DOI:** 10.1186/1471-2369-13-49

**Published:** 2012-06-28

**Authors:** Inger H Bleskestad, Harald Bergrem, Anders Hartmann, Kristin Godang, Lasse G Gøransson

**Affiliations:** 1 Department of Medicine, Stavanger University Hospital, Stavanger, Norway; 2 Section of Nephrology, Department of Medicine, Oslo University Hospital, Rikshospitalet, Oslo, Norway; 3 Section of Endocrinology, Medical Department, Oslo University Hospital, Rikshospitalet, Oslo, Norway

**Keywords:** Active vitamin D, Chronic kidney disease, Fibroblast growth factor 23, FGF23, Phosphate-binder, Parathyroid hormone, PTH, Sevelamer carbonate

## Abstract

**Background:**

Fibroblast growth factor 23 (FGF23) is a phosphaturic hormone that is secreted from bone and serum level increases as renal function declines. Higher levels of FGF23 are associated with increased mortality in hemodialysis-patients and in patients with chronic kidney disease (CKD) stage 2-4. The use of active vitamin D and phosphate binders as recommended in international guidelines, may affect the level of FGF23 and thereby clinical outcome. We investigated the effects of a phosphate binder and active vitamin D on the serum levels of intact FGF23 (iFGF23) and intact parathyroid hormone (iPTH) in patients with CKD stage 3b (glomerular filtration rate (GFR) 30–44 ml/min/1.73 m^2^).

**Methods:**

Seven women and 14 men were included, mean age 65.6 ± 12.2 years. They were randomized in a 1:1 ratio to receive one of two treatment sequences. Group-1 (the alphacalcidol-sevelamer carbonate group): alphacalcidol 0.25 μg once daily for two weeks followed by sevelamer carbonate 800 mg TID with meals for two weeks after a two-week washout period. Group-2 (the sevelamer carbonate-alphacalcidol group): vice versa. Nineteen patients completed the study. The 25-hydroxyvitamin D level at baseline was 97.6 ± 25.0 nmol/l.

**Results:**

There were no treatment effects on the iFGF23 and iPTH levels overall. In group-1 the iFGF23 level was higher after treatment with alphacalcidol compared with sevelamer carbonate (mean 105.8 ± 41.6 vs. 79.1 ± 36.5 pg/ml, p = 0.047 (CI: 0.4-52.9), and the iPTH level was lower (median: 26.5, range: 14.6-55.2 vs. median 36.1, range 13.4-106.9 pg/ml, p = 0.011). In group-2 the iFGF23 level increased non-significantly after treatment with sevelamer carbonate and throughout the washout period.

**Conclusions:**

In this crossover trial with alphacalcidol and sevelamer carbonate in patients with CKD stage 3b, the levels of iFGF23 were not significantly different after the two treatments. However, in the group of patients initiating therapy with sevelamer carbonate the iFGF23 levels seemed to increase while this response was mitigated in the group of patients given alphacalcidol followed by sevelamer carbonate. This may have therapeutic implications on choice of first line therapy. The number of patients is small and this conclusion is in part based on subgroup analysis. It is therefore important that these results are confirmed in larger studies.

**Trial registration:**

Trial Registration Number: European Clinical Trial Database (EudraCT) 2010-020415-36 and Clinical Trials.gov NCT01231438

## Background

Secondary hyperparathyroidism (SHPT) is a common complication of chronic kidney disease (CKD) and is characterized by abnormalities in serum calcium, serum phosphate, parathyroid hormone (PTH) concentrations, vitamin D metabolism, and bone turnover. SHPT is associated with increased cardiovascular disease, total mortality, and vascular calcifications [1]. Treatment guidelines for the evaluation and management of patients with chronic kidney disease-mineral and bone disorder (CKD-MBD) were published in 2009 [2] but there are to date no prospective trials documenting a survival advantage for any of the therapeutic treatment options available [3].

Phosphate retention occurs early in the course of renal failure and there are data in favor of the hypothesis that this is the principal abnormality of SHPT [4]. Fibroblast growth factor 23 (FGF23), a phosphaturic hormone, plays an important role in phosphate homeostasis. FGF23 secretion from bone increases as renal function declines [5]. FGF23 increases urinary phosphate excretion and can be viewed as an adaptive response to phosphate load. Klotho, a transmembrane protein, is mainly expressed in renal tubular cells, the parathyroid gland, and the choroid plexus and is an obligate co-receptor for FGF23 [6]. FGF23 inhibits 1,25-dihydroxyvitamin D (1,25 (OH)_2_D) production by inhibiting 1-α hydroxylase and stimulating 24-α hydroxylase thus acting as an anti-vitamin D agent. In CKD the expression of Klotho is reduced in the kidneys and also in the parathyroid glands [7]. This may explain the FGF23 resistance in parathyroid glands resulting in increased levels of both FGF23 and PTH in patients with renal failure [6].

Higher levels of FGF23 are associated with increased mortality in hemodialysis-patients even after correcting for phosphate levels and in patients with CKD stage 2–4 [8,9]. The use of active vitamin D and phosphate binders as recommended in international guidelines may affect the levels of FGF23 and thereby clinical outcome. We investigated the effects of a calcium-free phosphate binder and active vitamin D on the serum levels of intact FGF23 (iFGF23) and intact PTH (iPTH) in patients with CKD stage 3b.

## Methods

The study was designed as a single-center, randomized, open-label crossover study. Patients aged ≥ 18 years with CKD stage 3b (glomerular filtration rate (GFR) 30–44 ml/min/1.73 m^2^) were recruited from the nephrology outpatient clinic at Stavanger University Hospital. The study adhered to the Declaration of Helsinki and was approved by the Regional Medical and Health Research Ethics Committees (REC) Western Norway. The study was registered in the European Clinical Trial Database (EudraCT 2010-020415-36) and at Clinical Trials.gov NCT01231438. Written informed consent was obtained from all patients prior to inclusion.

Patients were excluded if they had had major surgery or a myocardial infarction within the last six months, were under active treatment for malignant disease, or any other clinically significant unstable medical condition. Patients who were on medical treatment with lithium, corticosteroids and/or bisphosphonates potentially influencing mineral metabolism were also excluded. None of the patients received treatment with any form of active vitamin D medication or phosphate binders prior to study start.

Twenty-one patients were included, seven women and 14 men, mean age 65.6 ± 12.2 years. They were randomized in a 1:1 ratio to two treatment groups, Table1. Group-1 received alphacalcidol (Etalpha®) 0.25 μg once daily for two weeks followed by two weeks washout and then sevelamer carbonate (Renvela®) 800 mg TID with meals (the alphacalcidol – sevelamer carbonate group). Group-2 received sevelamer carbonate (Renvela®) 800 mg TID with meals followed by two weeks washout and then alphacalcidol (Etalpha®) 0.25 μg once daily for two weeks (the sevelamer carbonate – alphacalcidol group). Doses were not adjusted during the treatment periods. The levels of 25-hydroxyvitamin D (25(OH)D) were analyzed before inclusion, and if 25(OH)D levels were < 40 nmol/l, they were given ergocalciferol (vitamin D_2_) 1.5 mg (two tablets of AFI-D_2_ forte® á 0.75 mg equivalent of 60 000 IU vitamin D_2_) every week for four weeks (a total of 8 tablets) and their 25(OH)D levels were then re-analyzed. If 25(OH)D levels were < 50 nmol/l but > 40 nmol/l they were given a total of 6 tablets of AFI-D_2_ forte® over three weeks. They were included when the 25(OH)D levels were > 50 nmol/l.

**Table 1:** Patient characteristics at inclusion

	**Total N = 21**	**Group - 1: alphacalcidol first N = 11**	**Group - 2: sevelamer carbonate first N = 10**	**p-value**
Age (years)	65.6 ± 12.2	65.7 ± 9.6	65.5 ± 15.1	0.98^a^
Sex (males, females)	14 - 7	7 - 4	7 - 3	0.76^b^
Body mass index (kg/m^2^)	26.4 ± 3.4	25.8 ± 4.0	27.1 ± 2.7	0.40^a^
SBP (mmHg)	132 (115–194)	132 (115–194)	133 (123–167)	0.89^c^
DBP (mmHg)	84 (76–114)	85 (78–114)	84 (76–99)	0.57^c^
Creatinine (μmol/l)	154.4 ± 25.5	149.9 ± 27.7	159.4 ± 23.3	0.41^a^
eGFR (ml/min/1.73 m^2^)	36.6 ± 5.6	37.6 ± 6.5	35.6 ± 4.6	0.44^a^
Albumin/creatinin ratio (mg/mmol)^d^	1.4 (0.2-95.0)	1.7 (0.2-95.0)	1.1 (0.3-32.0)	0.83^c^

Mean ± SD for normally distributed data, otherwise median and range.

^a^t-test for normally distributed variables.

^b^chi-squared test for categorical variables.

^c^Mann-Whitney U-testing of non-parametric continuous variables.

^d^measured at study end.

In Norway, the patients with CKD stage 3b are not given specific dietary advice. The phosphate content of beverages and phosphate additives in addition to the protein content of the diet is of utmost importance for the renal patients at risk of hyperphosphatemia [10]. Restrictions in intake are not advocated at this level of renal function, and since the study was carried out in a way to mimic standard clinical care, no advice was given other than asking the patients to continue with their usual diet and keep it stable during the study period. Blood samples were drawn in the morning after breakfast. A fasting morning urine specimen was delivered at every visit.

Compliance was monitored by pill counts. Two patients stopped taking sevelamer carbonate due to gastrointestinal side effects, one due to reflux symptoms, one due to constipation. These two patients were excluded from the analysis of the crossover study as a whole. Groups were otherwise unchanged as the randomization was not stratified and there was no reason to believe that the effect of the medication and the type of side effects were coupled in any way. The remainder of the patients took 93% of the prescribed doses of sevelamer carbonate. None of the patients experienced side effects of alphacalcidol and they took 99% of the prescribed doses. Two patients (one in each treatment group) had a washout period of four weeks due to winter holidays.

Patient characteristics: The underlying causes of CKD were hypertension (n = 6), adult polycystic kidney disease (n = 6), glomerulonephritis (n = 4) and unknown (n = 5). All but four patients were on antihypertensives: five patients on β-blockers, three on combined α- and β blockers, six on angiotensin-converting enzyme (ACE) inhibitors, 11 on angiotensin II (ARB) receptor blockers, six on calcium channel blockers, four on loop-diuretics, six on thiazid-diuretics, and two patients on spironolactone. Nine patients were on platelet inhibitors, 13 patients on cholesterol- lowering therapy (statins 12 patients, ezetimibe one patient), and six patients were on prophylactic anti-gout medication (xanthine oxidase inhibitors 5 patients, febuxostat one patient).

CKD stage 3b was defined as estimated GFR (eGFR) 30–44 ml/min/1.73 m^2^ estimated by means of the modification of diet in renal disease (MDRD) method [11]. White blood cell count and concentrations of hemoglobin, creatinine, total serum calcium, phosphorus, total alkaline phosphatase and albumin were determined at every visit using a computerized auto-analyzer. Serum and EDTA-plasma were then stored at −72°C for later measurements of iFGF23, iPTH, 25(OH)D and 1,25(OH)_2_D. Samples for markers of bone turnover: cross-linked N-telopeptides of bone type I collagen (NTx) as a bone-resorption marker and osteocalcin, bone-specific alkaline phosphatase (BALP) and intact N-terminal propeptide of type I procollagen (PINP) as bone-formation markers were stored in the same manner. These samples were then analyzed in one run in order to minimize analytical variability. All samples were measured in duplicate, with serial samples from a given individual run at the same time. Intra- and inter-assay coefficient of variation were <10% for all assays. Serum iFGF23 was measured by sandwich ELISA (Kainos Laboratories Inc., Tokyo, Japan), plasma iPTH by a radioimmunoassay (Scantibodies Laboratory, Inc., CA, U.S.A.) and serum 25(OH)D (DiaSorin, Minnesota, U.S.A.) and plasma 1,25(OH)_2_D (Immunodiagnostic Systems Nordic, Denmark) were quantified by radioimmunoassay (RIA). NTx in serum was determined by an enzyme-linked immuno-sorbent essay (ELISA/EIA, Wampole Laboratories Inc. Princeton, USA), as were serum osteocalcin and BALP (EIA, Quidel Corp. San Diego, USA). PINP was determined by RIA from Orion Diagnostica, Finland.

Urine samples were analyzed for concentrations of phosphate and creatinine at every visit and albumin at the last visit. The fractional excretion of phosphate (FePO_4_) was calculated as follows: (urine phosphate*serum creatinine)*100/(serum phosphate*urine creatinine).

### Statistical analysis

The primary end-points of the study were the differences in the levels of iFGF23 and iPTH after each treatment period. The secondary end-points were the differences in the levels of FePO_4_, calcium, phosphate, vitamin D metabolites and markers of bone turnover after each treatment period. The tests for period effects and carry-over effects were performed before the treatment effects were examined for the crossover study as a whole [12]. The two-sample t approach was then used to analyze treatment effects by comparing the period-differences (level after period 1 minus level after period 2) of the different end-points for normally distributed data otherwise the Mann–Whitney *U* test for independent samples was used [13]. The repeated measurement analysis of variance (ANOVA) was used to investigate changes over time for normally distributed data. When significant differences were found, appropriate t-tests were performed to obtain difference estimates and confidence intervals. When analyzing the differences over each period for the two groups separately, the paired samples *t*-test was used for normally distributed data, otherwise the Wilcoxon signed rank test for related samples was used. Differences were considered statistically significant at a p level of ≤ 0.05. All analyses were performed using the PASW Statistics version 18.0 (SPSS Inc, Chicago, IL).

### Power analysis

The primary end point, the difference in FGF23 levels between the two treatment periods in the two groups, would be analyzed with an independent two-sample *t* test. We found it reasonable to hypothesize that sevelamer carbonate therapy could bring the FGF23 levels in early CKD back to the range seen in healthy individuals. In dialysis patients, intravenous calcitriol therapy increased FGF23 levels and the increase was dose-dependent [14]. A conservative estimate of the alphacalcidol effect on FGF23 levels would thus be zero. The treatment period difference was then estimated to be 32 ng/ml [15]. We did not have any estimate of the standard deviation (SD) of the difference, but this is often lower than the SD of the measurements. We therefore chose to use the SD of the measurements as a conservative estimate of the SD of the difference [16]. With Δ 32, SD 24 and α 0.05 the power was 80% with 10 patients in each group.

## Results

The iFGF23 levels at baseline were > 50 pg/ml for all but one patient, Table2 (range in healthy adults 8.2 – 54.3 pg/ml [17]).

**Table 2:** Biochemical parameters for all patients at baseline (week 0), after the first treatment period (week 2), after washout (week 4) and after the second treatment period (week 6)

	**Group**^**a**^	**Baseline**	**After the first treatment period**	**After washout**	**After the second treatment period**
calcium (mmol/l) mean ± SD	1	2.33 ± 0.10	2.34 ± 0.11	2.35 ± 0.08	2.33 ± 0.07
	2	2.28 ± 0.07	2.30 ± 0.12	2.31 ± 0.11	2.34 ± 0.11
phosphate (mmol/l) mean ± SD	1	1.08 ± 0.24	1.11 ± 0.24	1.16 ± 0.20	1.11 ± 0.19
	2	1.03 ± 0.21	1.01 ± 0.18	1.04 ± 0.17	1.05 ± 0.19
creatinine (μmol/l) mean ± SD	1	141.1 ± 29.7	143.4 ± 27.1	138.9 ± 29.1	146.6 ± 35.1
	2	156.9 ± 26.8	166.2 ± 24.7	158.3 ± 21.6	169.5 ± 29.6
eGFR (ml/min/1,73 m^2^) mean ± SD	1	40.1 ± 7.0	38.9 ± 6.0	40.8 ± 7.7	38.9 ± 7.7
	2	36.4 ± 5.0	33.8 ± 2.7	35.6 ± 3.3	33.5 ± 6.3
25(OH) vitD (nmol/l) mean ± SD	1	99.4 ± 23.7	94.9 ± 22.3	93.8 ± 26.1	86.1 ± 18.0
	2	88.0 ± 22.0	86.6 ± 25.6	84.4 ± 31.0	85.6 ± 30.6
FePO_4_ (%)^b^ mean ± SD	1	26.8 ± 6.7	33.5 ± 9.1	31.7 ± 11.6	29.3 ± 8.6
	2	34.5 ± 9.5	36.3 ± 10.5	38.1 ± 12.2	41.0 ± 11.7
1.25(OH)_2_ vitD (pmol/l) median, range	1	72.0 (23.0-112.0)	70.0 (29.0-130.0)	64.0 (32.0-94.0)	61.0 (32.0-108.0)
	2	65.5 (46.0-175.0)	56.6 (37.0-125.0)	70.0 (27.0-89.0)	60.5 (37.0-99.0)
FGF23 (pg/ml) mean ± SD	1	90.7 ± 28.4	105.8 ± 41.6	102.6 ± 33.1	79.1 ± 36.5
	2	110.2 ± 73.7	127.6 ± 76.6	142.3 ± 52.2	110.4 ± 41.7
PTH (pg/ml) median, range	1	35.9 (16.3-147.4)	26.5 (14.6-55.2)	35.0 (14.3-72.5)	36.1 (13.4-106.9)
	2	35.3 (22.6-73.9)	38.7 (21.2-61.0)	33.7 (23.6-63.1)	37.7 (19.7-71.3)
PINP (μg/l) median, range	1	56.5 (23.2-115.6)	53.0 (24.5-133.8)	52.8 (30.0-89.7)	60.8 (28.3-82.2)
	2	36.9 (17.1-55.6)	40.5 (20.3-61.4)	37.9 (17.5-55.4)	38.4 (16.8-49.2)
Osteocalcin (ng/ml) median, range	1	17.3 (7.6-37.7)	18.1 (8.9-38.7)	16.5 (9.4-35.6)	18.1 (9.6-34.9)
	2	12.8 (7.4-25.2)	13.5 (7.7-28.0)	13.6 (9.7-24.5)	13.4 (10.5-26.4)
BALP (U/L) median, range	1	24.3 (14.7-43.4)	22.8 (13.0-37.8)	25.1 (13.0-39.3)	27.1 (13.7-41.2)
	2	17.9 (12.7-32.0)	19.7 (10.8-33.0)	19.2 (9.4-35.1)	21.3 (11.6-36.6)
NTx (nM BCE)^c^ median, range	1	16.8 (10.3-77.0)	14.4 (10.4-54.8)	13.6 (10.2-25.6)	13.9 (11.1-42.2)
	2	13.2 (5.9-34.3)	11.9 (7.3-18.3)	11.8 (6.9-21.4)	14.1 (8.0-22.1)

^a^Group-1: The alphacalcidol-sevelamer group: alphacalcidol for two weeks (first treatment period) followed by sevelamer carbonate (second treatment period) after two weeks washout (data shown for the nine patients that completed the crossover study).

Group-2: The sevelamer-alphacalcidol group: sevelamer carbonate for two weeks (first treatment period) followed by alphacalcidol (second treatment period) for two weeks after two weeks washout.

^b^The treatment difference was statistically significant (p = 0.028).

^c^nM BCE = nmol Bone Collagen Equivalents.

**Figure 1:**
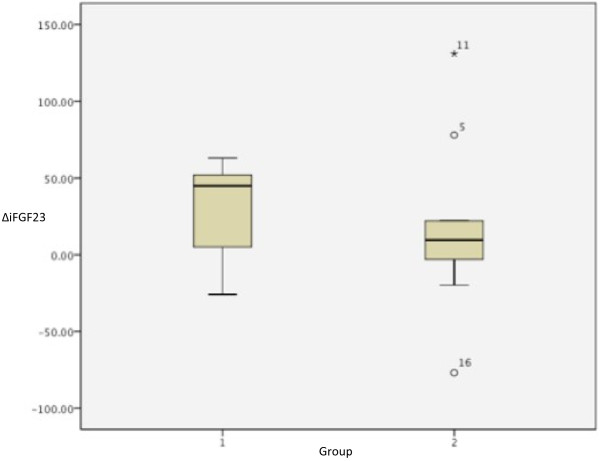
**Delta iFGF23 levels.** Box plot of Δ intact fibroblast growth factor 23 (iFGF23): mean iFGF23 level after the first treatment period minus mean iFGF23 level after the second treatment period. Group-1: the alphacalcidol – sevelamer carbonate group: alphacalcidol for two weeks (first treatment period) followed by sevelamer carbonate for two weeks (second treatment period) after two weeks washout. Group-2: the sevelamer carbonate – alphacalcidol group: sevelamer carbonate for two weeks (first treatment period) followed by alphacalcidol for two weeks (second treatment period) after two weeks washout.

There was no significant period or carry-over effect on the iFGF23 (p = 0.056 and p = 0.234) or iPTH levels (p = 0.315 and p = 0.315). There was no significant treatment effect on iFGF23 (Figure1) or iPTH levels (p = 0.667 and p = 0.243 respectively).

There was no significant period effect (p = 0.908) or carry-over effect (p = 0.109) on FePO_4_. There was a significant treatment effect with a higher urinary excretion of phosphate after two weeks of treatment with alphacalcidol compared with sevelamer carbonate (mean difference 4.4%, p = 0.028, CI: 0.6-8.3).

There was no treatment effect on the bone-resorption marker NTx, the bone-formation markers BALP, osteocalcin and PINP. Despite treatment with alphacalcidol, the 1,25(OH)_2_D levels were unchanged. There were no treatment effects on serum calcium and phosphate levels, Table2.

There was no difference in creatinine levels and eGFR measurements between the two groups at baseline or during the study. For the two groups combined the creatinine levels were significantly higher (mean difference 9.2 μmol/l, CI: 2.9-15.5, p = 0.007) and the eGFR levels were significantly lower (mean difference 2.1 ml/min/1.73 m^2^, CI: 0.5-3.7, p = 0.011) at the end of the study compared with the start of the study. The magnitude of the reduction in eGFR during the study although statistical significant, is unlikely to have had any effect on the result of the study. There was no difference in 25(OH)D levels between the two groups during the study. There was a trend toward lower levels at the end of the study compared with the earlier visits, but the trend was not statistically significant (p = 0.075).

### Within-groups comparisons

#### Group-1, the alphacalcidol – sevelamer carbonate group

The iFGF23 level was higher after treatment with alphacalcidol compared to sevelamer carbonate, (mean 105.8 ± 41.6 vs. 79.1 ± 36.5 pg/ml, p = 0.047 (CI: 0.4-52.9), for iPTH lower (median: 26.5, range: 14.6-55.2 vs. median 36.1, range 13.4-106.9 pg/ml, p = 0.011).

**Figure 2:**
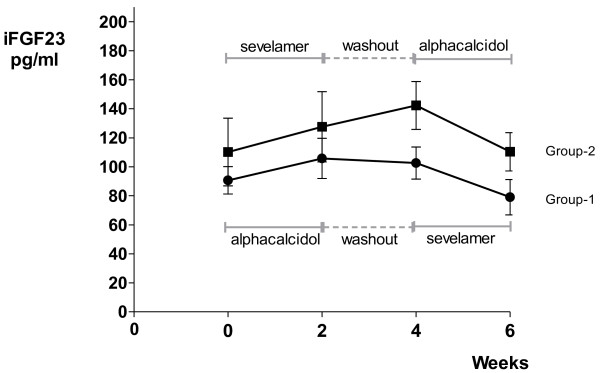
**Mean iFGF23 levels and study design.** The mean level of intact fibroblast growth factor 23 (FGF23) for each group at baseline (week 0), after the first treatment period (week 2), after washout (week 4) and after the second treatment period (week 6). Bars indicate SEM. Group-1: the alphacalcidol – sevelamer carbonate group: alphacalcidol for two weeks (first treatment period) followed by sevelamer carbonate for two weeks (second treatment period) after two weeks washout. Group-2: the sevelamer carbonate – alphacalcidol group: sevelamer carbonate for two weeks (first treatment period) followed by alphacalcidol for two weeks (second treatment period) after two weeks washout.

iFGF23 tended to be higher after treatment with alphacalcidol compared to baseline, but it did not reach clinical significance (p = 0.070), Figure2. FePO_4_ was significantly higher after treatment with alphacalcidol (mean difference 6.7%, CI: 2.3-11.1, p = 0.007). There was no effect of alphacalcidol on the iPTH levels (p = 0.083).

iFGF23 was significantly lower after treatment with sevelamer carbonate compared to the level before initiation of treatment (mean difference 23.4 pg/ml, CI: 1.4-45.5, p = 0.040). There was no effect of sevelamer carbonate treatment on FePO_4_ (p = 0.366) or the iPTH levels (p = 0.678), Figure1.

#### Group-2 the sevelamer carbonate – alphacalcidol group

The iFGF23 levels increased non-significantly after treatment with sevelamer carbonate (p = 0.169) and throughout the washout period (p = 0.074), Figure2. There was no effect of sevelamer carbonate on FePO4 (p = 0.597) or on the iPTH levels (p = 0.878).

There were no effects of alphacalcidol on the iFGF23 levels (p = 0.168), FePO4 (p = 0.482), or the iPTH levels (p = 0.284) when alphacalcidol followed sevelamer carbonate therapy.

The two patients with a washout period of four weeks responded to the two treatments in accordance with the rest of the patients in their group.

## Discussion

In this six-week crossover study in CKD patients stage 3b there were no treatment effects on the iFGF23 (Figure1) or iPTH levels.

The major effect of phosphate binders is to bind phosphate in the alimentary tract and thereby decrease phosphate absorption. Reduced phosphate absorption over five to nine days reduces the levels of FGF23 in healthy humans [18-21]. We therefore expected a reduction in iFGF23 levels after the intervention period with a phosphate binder. The baseline levels of serum phosphate and iPTH were however low in our cohort of patients indicating a low dietary intake of phosphate. The potential effect of a phosphate binder may therefore be reduced.

Our results are in accordance with a study of dietary phosphate restriction and therapy with lanthanum carbonate of two weeks’ duration in which the levels of FGF23 were not reduced [22]. However, this is in contrast to a four-week study with lanthanum carbonate and a six-week study of sevelamer hydrochloride where the levels of FGF23 were reduced [23,24]. The authors of the two-week study hypothesized that an intervention period of two weeks was too short to reduce the FGF23 levels. In the group of patients that received alphacalcidol as the first line of therapy in our study, the iFGF23 level was however significantly lower (p = 0.040) after two weeks of sevelamer carbonate treatment compared with the level before initiating therapy. This suggests that an intervention period of two weeks in itself is not too short to induce a reduction in iFGF23 levels even in CKD patients.

One of the strengths of this study is that it includes an active form of vitamin D in the treatment regiment and that the 25(OH) vitamin D levels at inclusion and during the study were sufficient. Active vitamin D enhances phosphate absorption from the alimentary tract through the up-regulation of the sodium-phosphate co-transporter, NaPi-IIb, in the small intestine, and increases renal phosphate reabsorption by inducing the renal sodium-phosphate co-transporter NaPi-IIc [25]. Infusion of 1,25(OH)_2_D increases FGF23 levels in normal mice [26] and in dialysis patients [14]. Active vitamin D exerts a direct effect on the parathyroid gland which leads to reduced excretion of PTH [6]. The net effect of vitamin D treatment is thus difficult to predict, and it is important to include this treatment regimen in clinical studies. The levels of calcium, iPTH and 1.25(OH)_2_D did not change during the study. This may be due to the relatively low dose of alphacalcidol given (0.25 μg once daily).

Although we did not perform bone-biopsies, which are the gold standard in assessing bone turnover, one of the strengths of the study is the characterization of markers of bone turnover. Either the bone-resorption marker or the bone-formation markers’ changed during the study indicating a steady state of bone turnover. Urinary phosphate excretion during a steady state of bone turnover is mainly determined by phosphate absorption from the alimentary tract [27]. The changes in urinary phosphate excretion during the study can thus be attributed to changes in phosphate absorption. The FePO_4_ was higher after treatment with alphacalcidol compared with sevelamer carbonate overall (mean difference 4.4%, p = 0.028, CI: 0.6-8.3) consistent with increased phosphate absorption.

Our study is a crossover study, and this design has its limitations. The validity of the results of a crossover trial depends on the study results only being affected either by treatment effects or chance. If there are interactions between period and treatment, the crossover study as a whole can no longer be analyzed in an ordinary way and one could be forced to exclude the results for the second treatment period for both groups.

The statistical testing for a period effect was negative for all the samples analyzed. The results for iFGF23 were on the other hand unexpectedly lower in the second period than in the first period in both groups (Figure2). But if a period effect was present, for example by increased dietary phosphate intake at Christmas-time, we would expect the levels of iFGF23 to increase in the second treatment period, not decrease.

The statistical testing for a carry-over effect was also negative for all the samples analyzed. When examining the results for each group separately (Figure2), one can see that the iFGF23 levels have not returned to their pre-treatment levels after the washout period. A carry-over effect could explain this finding thus indicating that the statistical test simply was underpowered due to small sample size. In a study with sevelamer hydrochloride, Oliveira et al showed that the iFGF23 levels did return to pre-treatment levels two weeks after study end [24]. In group-1, the iFGF23 levels tended to decrease during the washout period although they did not return to pre-treatment levels. This is in contrast to the iFGF23 levels in group-2 that continued to rise during the washout period. The patients were asked to continue with their diet unchanged and the dietary advice was the same for the two groups. It is thus unlikely that the rise in iFGF23 levels after sevelamer carbonate therapy in group-2 was a result of phosphate loading. There was also no significant change in FePO4 in these patients indicating a stable phosphate intake.

The difference between the two groups is striking. We suggest that the reason is an order effect that is clinically interesting and not a result of dietary changes or a treatment by period interaction (carry-over effect). The patients who received alphacalcidol first responded to sevelamer carbonate treatment with a reduction in iFGF23 levels as expected in contrast to patients receiving sevelamer carbonate first. This could be due to an unknown effect of active vitamin D mediated by the gastrointestinal tract. Data in favor of an “intestinal factor” involved in phosphate homeostasis is emerging [28]. It has been demonstrated that installation of phosphate in the duodenum of normal rats rapidly increased renal excretion of phosphate without accompanying FGF23 elevations [29]. In humans, intravenous infusion of phosphate increased serum phosphate levels but FGF23 levels did not change for up to six hours [30]. Meals containing 1200 mg inorganic phosphate increased FGF23 levels but not until after 8 hours [31]. The route of administration and the duration of the phosphate load appear to be of importance.

Despite small sample size and the limitations of the study due to the crossover design, the results of our study should be interesting for clinicians planning future studies. The questions raised also emphasize the need to include active vitamin D in studies evaluating treatment effects on iFGF23 levels.

## Conclusions

Our findings are challenging and suggest that treatment with sevelamer carbonate may have to be preceded by alphacalcidol in order to reduce iFGF23 levels during the initial phase of treatment. Alphacalcidol given alone may on the other hand increase iFGF23 levels. This may have therapeutic implications and needs confirmation in larger studies.

## Competing interests

IHB has received lecture fees or travel funding from Shire, Pfizer, Novartis and Eli Lilly. HB has received travel funding from Amgen and Roche. LGG has received lecture fees or travel funding from Genzyme, Novartis, Amgen, Swedish Orphan and Abbot.

## Authors’ contributions

IHB participated in the design of the study, collection of the data, performed the statistical analysis and made substantial contributions to the interpretation of data and drafted the article. HB has participated in the interpretation of the data and revising the manuscript. AH has participated in the interpretation of the data and revising the manuscript. KG carried out the immunoassays and made contributions to the methods section. LGG has designed the research, made substantial contributions to the statistical analysis and interpretation of data, helped to draft the manuscript and revised it. All authors read and approved the final manuscript.

## Pre-publication history

The pre-publication history for this paper can be accessed here:

http://www.biomedcentral.com/1471-2369/13/49/prepub
